# Effect of Dietary Enrichment with Flaxseed, Vitamin E and Selenium, and of Market Class on the Broiler Breast Meat—Part 2: Technological and Sensorial Traits

**DOI:** 10.3390/foods11172567

**Published:** 2022-08-25

**Authors:** Ambrogina Albergamo, Rossella Vadalà, Daniela Metro, Daniele Giuffrida, Francesco Monaco, Stefano Pergolizzi, Michelangelo Leonardi, Giovanni Bartolomeo, Massimiliano Petracci, Nicola Cicero

**Affiliations:** 1 Department of Biomedical, Dental, Morphological and Functional Images Sciences (BIOMORF), University of Messina, Viale Annunziata, 98100 Messina, Italy; 2 Science4Life Srl, an Academic Spin-Off, c/o BIOMORF Department of University of Messina, Viale Annunziata, 98100 Messina, Italy; 3 Department of Agricultural and Food Sciences (DISTAL), Alma Mater Studiorum, University of Bologna, Piazza Goidanich 60, 47521 Cesena, Italy

**Keywords:** poultry, breast meat, enriched fed, physicochemical properties, water holding capacity, sensory analysis

## Abstract

The influence of diet enrichment with flaxseed, selenium and vitamin E, and market class on breast meat was investigated in terms of technological and sensorial quality of breast meat. A randomized complete block design with an experimental unit of *n* = 6000 broilers receiving a standard or enriched diet, and slaughtered at 37 (light class), 47 (medium class), or 57 (heavy class) days of life, was developed. Then, enriched and standard breast muscles from every market class were studied for their technological and sensorial traits—both at 24 h post-mortem and after one month of frozen storage—by a statistical multiple linear model. Redness and yellowness of muscles significantly (*p* < 0.05) increased and decreased with increasing market age. Moreover, the yellowness significantly (*p* < 0.05) raised after frozen storage. However, obtained data were always indicative of a normal meat color. The water holding capacity improved following fed enrichment and significantly (*p* < 0.05) worsened after frozen storage. For the sensory analysis, juiciness and chewing rest of meat resulted significantly (*p* < 0.05) improved with increasing slaughtering age and diet enrichment, as well as their mutual interaction, while they deteriorated after frozen storage. Overall, fresh and enriched muscles from heavy broilers had the best technological and sensorial traits, thus, confirming that market size and diet should be highly considered to obtain breast meat with greater consumer acceptance.

## 1. Introduction

As a matter of fact, the poultry meat segment has been exhibiting the most noticeable growth rate over the other types of meat. According to the estimates from the OECD-FAO, the global consumption of meat is projected to rise by 14% over the next decade, compared to the average of 2018–2020. Meat availability from poultry, sheep, pork, and beef meat is estimated to grow respectively 17.8%, 15.7%, 13.1%, and 5.9% by 2030. Worldwide, poultry will presumably account for 41% of all the meat sources in 2030 (+2% when compared to the years 2018–2020), the quotas of other meat products being lower, i.e., beef (20%), pork (34%), and sheep (5%) [1]. On this basis, the global meat consumption is estimated to grow to 35.4 kg per capita in the next decade, of which over one-half of this increase is attributed to poultry. The shift towards poultry consumption is mainly related to the precious nutritional value of such meat, its desired sensory properties, the relative affordability, and the versatility showed not only in the home, but also in the manufacturing of a variety of processed goods. Other driving forces are represented by the absence of cultural/religious constraints to its consumption, as well as the ability of the production system to promptly research higher standards in meat and to adapt to the evolution of consumer expectations [2,3]. In this regard, the production system, whether conventional, free-range, or organic, with its key factors such as feeding regimen and (pre) slaughter conditions, may differentially affect the meat quality, and consequently, its market price [4,5].

In Italy, a conventional system based on the indoor rearing of fast-growing broilers derived from intensive selection practices, i.e., commercial hybrids-, as well as on optimized feeding programs and housing conditions is mainly adopted. The production system also relies on the production of broilers with three different market ages by separately rearing female and male chicks during specific time periods. In this respect, light-size birds (i.e., 32–37 day old females weighing 1.2–1.7 kg) are for rotisserie products, medium-size birds (42–49 day old females or males reared up to 2.3–2.8 Kg) are mainly reserved for cut-up products, and heavy-size broilers (50–60 day old males with a weight of 3.2–4.2 Kg) are produced for cut-up and further processed meat derivatives, and they are generally preferred by the large-scale retail trade [6].

In our recent study [7], we investigated how market class and feeding program—which included functional ingredients such as extruded flaxseed meal (FSM), vitamin E, and selenium (Se) could affect the quality of breast meat in terms of nutritional and functional properties. However, the study of the technological and sensorial profile is also of utmost importance, especially for the poultry industry, because the broiler meat is nowadays mainly consumed as cuts (available estimates: 40% and 48%, respectively in US and France) and processed products (available estimates: 49% and 29%, respectively in US and France) and the underestimation of such aspects may create a bad image of meat products, thus, unavoidably causing a lack of confidence in clients and consumers and compromising the success of products [3,8]. Technological quality includes a variety of physicochemical traits of meat, such as pH, color, and water-holding capacity (i.e., drip loss and cooking loss), whereas the sensorial profile is generally depicted by attributes such as overall appearance, taste, and texture of meat. Both technological and sensorial traits reflect the ability of meat to be processed, its stability after refrigerated or frozen storage, as well as its acceptance degree [9,10,11], and they may strictly depend, among other factors, on the market class and dietary strategy.

While only a previous work [6] evaluated the effect of market size on the technological attributes of breast muscles at 24 h post-mortem and concluded that the slaughtering age of broilers may remarkably affect meat quality, different studies explored the technological and sensorial traits of breast muscles, both fresh and after storage, from broilers receiving diets with flaxseed, Se, and vitamin E, alone or in combination [12,13,14,15,16,17]. Indeed, a flaxseed-based diet program notoriously enhances the lipid profile of chicken meat- especially in terms of polyunsaturated fatty acids, but may negatively affect its storage stability, in terms of WHC, texture, and flavor, due to the increased unsaturation degree contributing to the lipid oxidation [18]. Hence, the inclusion in feed of antioxidant molecules, such as vitamin E and Se, may ameliorate not only the healthiness of the product but also its technological and sensorial traits, even after prolonged storage, with a final higher consumer acceptance [17].

With such a background, aim of the study was to study the effect of market age and functional feeding with flaxseed, selenium and vitamin E, as well as their interdependence, on the technological and sensorial traits of chicken breast muscles both at 24 h post-mortem and after one month of frozen storage.

## 2. Materials and Methods

### 2.1. Production System and Diet Programs

The experimental design of the study was already described in our previous study [7]. Briefly, a total of 36,000 of 1-day old and mixed sex broiler chicks (breed: Ross 308) were farmed and slaughtered in dedicated plants of the company “Avimecc S.p.A.” (Ragusa, Italy) during June–July 2021. The birds were placed into 72 flocks (500 birds/flock) intended to produce light- (*n* = 24), medium- (*n* = 24), and heavy- (*n* = 24) sized chickens and they were reared for 6–8 weeks, depending on the market class.

All flocks were randomly assigned to the conventional (*n* = 36, equally split into light-, medium-, and heavy sized broilers) and experimental (*n* = 36, equally split among light-, medium-, and heavy sized broilers) feeding programs. Broilers were continuously provided drinking water, and a starter diet was provided for the first 10 days of life, a grower diet from day 10 up to day 24, another grower diet up to day 39, and a finisher diet was fed until the end of the trial period.

The ingredients of both conventional and experimental diets, as well as their proximate composition, are shown in Table 1.

At pre-established commercial ages, five chickens per flock were randomly chosen and slaughtered under commercial conditions. Specifically, light broilers (*n* = 120, equally split between birds receiving conventional diet and birds adopting functional diet) were slaughtered at 37–40 days of life, with a live weight of 1.7–2.0 kg; medium broilers (*n* = 120, split as described above) were slaughtered at 45–48 days of life, with a live weight of 2.3–2.8 kg; while heavy broilers (*n* = 120, split as described above) were slaughtered at 54–57 days of life, with a live weight of 3.0–3.5 kg.

All experimental practices were carried out following the ethical principles of the Council Directive 2007/43/EC [19].

### 2.2. Samplesa

Boneless, skinless, breast muscles from every market class of broiler intended for functional (*n* = 60 split into equal triplicates) and conventional (*n* = 60 split as described above) feeding were obtained at 3 h post-mortem. Subsequently, the breast fillets were transported to the laboratory in polyethylene bags and under refrigerated temperature.

**Figure 1:**
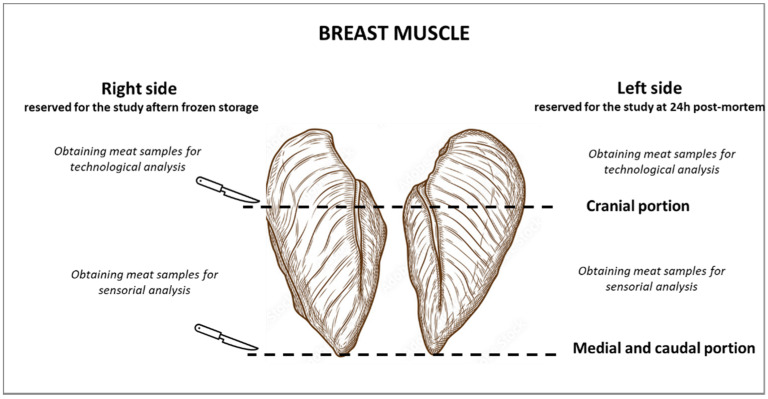
Sampling of the breast muscle in relation to the analyses of the study.

In the laboratory, every fillet was trimmed of excess fat and connective tissue and cut in its left and right halves, which were considered as independent samples. The final doubling of samples was necessary to ensure that a single breast muscle received all the analyses required by the study, both in the fresh state and following a prolonged frozen storage (Figure 1). In fact, the left side was divided into the cranial portion, which was stored at 4 ± 0.5 °C until forthcoming analysis of the technological traits, and into the medial and caudal part, which was kept at 4 ± 0.5 °C (EVERmed chiller, EVERmed S.r.l., Mantova, Italy) up to the imminent sensorial analysis. On the other hand, the right half was selected for the study of the technological (cranial part) and sensorial (medial + caudal portion) properties of the frozen breast fillet stored at −18 ± 1 °C (Desmon freezer, Desmon S.p.A., Avellino, Italy) for 30 days (Figure 1). For the frozen samples, both technological and sensory analyses were preceded by thawing overnight muscles at 4 ± 0.5 °C.

### 2.3. Technological Properties

Harmonized procedures already described by Petracci and Baéza [20] were employed for studying the technological potential of conventional and functional breast fillets, both at 24 h post-mortem and after 30 days of frozen storage. Briefly, the intensity of the acidification fall in the breast muscle was measured in a portion of the cranial part of every sample (~1 g) by employing a digital portable pH-meter (model 3510, Jenway, United Kingdom). Briefly, the pH was determined by placing the electrode into the muscle following its incision. The electrode was calibrated at pH 4.0 and 7.0 every 30 measurements.

A portable colorimeter (CR-400, Minolta, Tokyo, Japan) with pulsed xenon arc lamp, average daylight illuminant D65, a standard observer at 10°, and CIE L*a*b* color scale, was exploited to measure the color of the bone-side face of each sample. In the color determinations, only sample areas free of color defects (i.e., hemorrhages, bruises, blood vessels etc.,) were taken into consideration.

The WHC was measured in terms of drip loss and cooking loss. For the drip loss, part of the cranial portion of every chicken breast (~50 g) was weighed (W1), kept hanging within a polyethylene bag during 48 h at +4 °C (EVERmed chiller, EVERmed S.r.l., Mantova, Italy), and finally weighed again (W2). Hence, the drip loss (%) was gravimetrically determined by the difference between W1 and W2. For the cooking loss, another part of the cranial portion of breast muscle was vacuum packaged and cooked in a water bath at 80 °C for 45 min until an internal core temperature of 80 °C was achieved. Then, it was re-weighed to determine gravimetrically the cooking loss (%).

### 2.4. Sensorial Traits

The effect of the feeding manipulation on the organoleptic quality of the breast fillet from different market classes was assessed by considering fresh meat (24 h post-mortem) and frozen meat stored for 30 days. To this purpose, a descriptive sensory analysis was conducted according to the standards of the ISO 13299:2016 [21]. A sensory panel of 7 members (4 females and 3 males ranging from 30 to 60 years old) was set up with professors, researchers, and technicians of the University of Messina with a strong background in the food area, including the sensorial analysis of meat, and trained according to the criteria of ISO 8586:2012 [22].

First, fresh fillets as well as thawed fillets derived from the frozen storage, were presented in white dishes to evaluate attributes such as the appearance (intended as a combination of color pleasantness, textural uniformity, and general structure), the tactile sensations (i.e., slickness and tackiness), and the odor (overall) [23].

Then, the same samples were cooked 5 min per side by employing a pre-heated non-stick pan, so that a final internal core temperature of 80 °C was assured, and immediately served in white dishes for assessing attributes such as the visual aspect (i.e., combination of color pleasantness, textural uniformity, and general structure), the flavor (overall), the taste (overall), and the texture (i.e., tenderness, juiciness, and chewing rest, intended as the meat residue in mouth at the swallowing time) [24]. A linear and non-structured scale going from 0 (no intensity) to 8 (high intensity) was employed to rate every attribute.

Sensory tests were carried out both on raw and cooked samples in a testing room conditioned to a temperature of 21 ± 2 °C, equipped with several individual chambers having wall and furniture of neutral color, and standard lighting. Every panelist conducted the sensory test in a booth and had no specific information about the samples.

### 2.5. Statistical Analysis

For every market class, the number of flocks aimed to conventional (*n* = 12) or functional (*n* = 12) feeding, represented the experimental unit for all the obtained data. Consequently, technological data were given as mean ± standard deviation of *n* = 60 replicate measurements per experimental unit. However, due to time and practical constraints of sensory analysis, the relative data were provided in terms of mean ± standard deviation of *n* = 7 replicate measurements per experimental unit.

Hence, a Shapiro–Wilk test was run to verify the initial assumptions of experimental data. Then, the GLM procedure of the SAS statistical package (SAS Institute Inc., Cary, NC, USA) was employed to analyze data according to a multiple linear regression as already described by Albergamo and colleagues [7]. The model included data from breast muscles as dependent variables, and the diet program, the market size, the storage, and their mutual interaction as fixed factors. Statistical significance was accepted with *p* < 0.05. For statistically significant relations of investigated parameters with the market class, significant differences among the mean values were checked by a post-hoc Tukey’s honestly significant difference (HSD) test. However, for the comparison of a pair of independent treatments (i.e., type of diet and storage process), a *t*-test was conducted. The statement of significance was set at *p* < 0.05.

## 3. Results and Discussion

### 3.1. Technological Traits

The technological properties of standard and enriched pectoral muscles from light, medium, and heavy chickens are shown in Table 2.

#### 3.1.1. pH

The extent of decrease in pH observed in post-mortem muscle notoriously affects both processing ability and sensorial quality of poultry meat [25,26,27]. Generally, the normal pH of chicken breast meat measured at 24 h post-slaughter (ultimate pH, pHu) varies between 5.0 and 6.0. A low pHu results in “acid meat” (<5.7), with similar defects to those of PSE (pale, soft, exudative) meat with a pale aspect and reduced WHC, while high ultimate pH (>6.0) leads to dark, firm, and dry meat with poor storage quality [28,29].

In the present study, all considered variables (i.e., market class, feeding program, and the frozen storage) did not significantly impact the glycolytic metabolism of muscle post-mortem and, as a result, the mean values of pH were within the normal range described above. The pH increased, although not significantly, with increasing market age (from 5.81 to 5.92, *p* > 0.05) (Table 2). Berri and colleagues [30] proposed that the increase in fiber cross-sectional area due to the increased size of birds at slaughter may be associated with higher ultimate pH values. Indeed, an increased cross-sectional area of the muscle fiber corresponded to an enhanced muscle creatine kinase activity and related shifts in tissue metabolites, due to the reduction of the glycolytic pathway and lactic acid levels [30]. As expected, the pH did not significantly change in products obtained from standard and enriched diets (5.86 and 5.85, *p* > 0.05), and slightly decreased in meats frozen stored for 30 days (5.89 to 5.82, *p* > 0.05) (Table 2). With respect to the latter variable, since the frozen storage and the subsequent thawing cause the loss of water, they may lead to a concentration of the solutes, responsible for lower pH values in breast fillets [31]. The linear model highlighted that none of the variables investigated, nor their mutual interaction, impacted such parameter (Table 2).

In agreement with the findings from this study, previous studies already suggested that the pH increased with age at slaughter [6,32]. Similarly, Dal Bosco and co-workers reported that pH of breast muscles from broilers (genotype Naked Neck, CN1) was lower at 70 days of life than 81 days [33]. Połtowicz and colleagues pointed out a rising pH in the breast from chickens with 35 and 42 days of life [34]. Nevertheless, Janischand colleagues demonstrated that pH was not affected by slaughtering age [35]. Literature also reported that the pH of breast meat did not significantly change in relation to the functionalization of feed with flaxseed for increasing time periods [36,37], with different forms of Se [13,38], as well as with Se and vitamin E, alone or in combination [17,39]. With respect to the storage, the pH of breast meats confirmed to decrease after the prolongation of frozen storage [31,40,41,42]. However, Soglia and colleagues [43] did not find any variation in pH by comparing the values assessed on fresh and frozen/thawed broiler meat in agreement with previous findings in lamb and horse meat [44,45].

#### 3.1.2. Color

Meat color is generally chromatically correlated to the amount of heme-containing compounds, such as myoglobin and its forms, and its variation, especially in terms of hue and chroma, may be due to a variety of intrinsic and extrinsic factors affecting the content of the heme pigment, i.e., iron [46]. Besides that, meat color is also determined achromatically by the extent of light scattering of the meat microstructure and its change, in terms of lightness, is highly affected by post-mortem muscle events [47]. In the specific case of poultry meat, the color is influenced to a lesser degree by the content of heme pigment and other intrinsic (i.e., slaughtering age, sex, and genotype of birds) and extrinsic (i.e., diet and rearing system) variables have certainly a greater impact [48].

In the color measurement, lightness (L*) determination may be intended as an indicator of breast meat quality for further processing [49]. Generally, a L* value comprised between 50 and 56 is indicative of normal breast muscles, while L* < 50 and L* > 56 respectively of dark and pale breasts [49]. In the present study, L* values were within the normal range, when considering all the variables investigated. However, concerning the market class, L* first increased in products from light to medium birds and then decreased in those from heavy broilers, although not statistically significant (respectively, 53.17, 54.28, and 52.60, *p* > 0.05) (Table 2). Additionally, the negative relation between pH and L* of broiler breasts, already reported in literature [50], was confirmed in the present study, as the darkest fillets from heavy bird exhibited the highest pH. This may be explained by the fact that a higher pH than the isoelectric point of the myofibrillar proteins (5.0) would induce water molecules to be tightly bound to the myofibrils and, therefore, a better water retention capacity and meat color as well [29,51]. According to the dietary strategy, non-significant differences were observed between standard and enriched products (respectively, 53.47–53.22, *p* > 0.05), while fresh fillets were darker than those freeze-stored for 30 days (54.13–52.57, *p* > 0.05) (Table 2). The discoloration observed in breast samples after frozen storage may be related to the degradation of myoglobin forms, such as deoxymyoglobin and oxymyoglobin, by oxidation and reduction reactions, as well as to the loss of water which, as already described above, would lead to lower pH of meat [52]. As a result, the inverse relationship between pH and L* observed in fresh products was not met in frozen fillets. According to the statistical analysis, none of the studied independent factors, nor their mutual interaction, affected the lightness of breast muscles (Table 2).

In breast meat, it is well-known that the redness (a*) is inversely related with L* and directly related with the tissue pH, such that a higher redness is generally observed in darker breasts with higher pH [6,53]. In the present study, this occurred when considering the market class, and only partially in relation to frozen storage. In fact, a* was not significantly different in light and medium breasts, and then, significantly increased in products from heavy chickens (respectively, 2.47, 2.55, and 2.97, *p* < 0.05), which were characterized by lower L* and higher pH values. With respect to the storage, frozen/thawed breasts with lower L* and lower pH, showed a higher a* value than fresh meats with higher L* and pH (respectively, 3.28 and 2.04, *p* < 0.05). However, no relation between L*, pH, and a* was highlighted in standard and enriched products, which, moreover, turned out to be non-significantly different in terms of redness (respectively, 2.67 and 2.65, *p* > 0.05). The multiple linear model revealed that the a* of breast muscles was significantly affected by market class, as well as by all the possible interactions among the considered fixed factors (Table 2).

The literature has reported that the yellowness (b*) of breast meat decreases together with L* as the pH and a* increase [26]. This means that chicken meat with a high pH is generally darker and redder in color. On the other hand, paler meat with lower pH is related to higher b* values [29,46]. These relations were supported by breast fillets with different market ages. In fact, the yellowness significantly varied from 5.42 to 4.77 (*p* < 0.05), when moving from light to heavy products with concurring characteristics of pH, L*, and a*. However, the same could not be said for frozen/thawed meat, as b* was equal to 4.43 and 5.17 (*p* < 0.05) in paler and redder/darker products, respectively. Additionally, no significant changes were observed in conventional and functional muscles which showed the same yellowness degree (4.81 and 4.79, *p* > 0.05). The statistical model revealed a significant impact of single variables investigated, and of their mutual interactions as well, in determining the yellowness of various meat samples.

A literature overview suggested contrasting color measurements of breast muscles from broilers with increasing age. Bianchi and colleagues focused on broilers from the Italian production system and revealed decreasing L*, a*, and b* values when moving from light to heavy breast fillets [6]. Conversely, Dal Bosco et al. found out increasing lightness, redness, and yellowness in breasts of chickens from different genotypes going from 70 to 81 days of life [33]. On the other hand, Połtowicz and colleagues highlighted a growing trend for L* and decreasing trends of a* and b* in breasts from chickens from 35 to 42 days of life [34]. With respect to the feeding program, conclusions mostly consistent with data from our study were achieved. In fact, Kumar and colleagues revealed no significant effects on the color of breast muscles from broilers fed flaxseed at increasing time durations [37]. Nevertheless, Miezeliene and coworkers detected a decreased lightness (L*), and increased redness (a*) and yellowness (b*) as an effect of an increasing Se supplementation in diet [14]. Values of L*, b*, and a* of breast muscles from broiler receiving various Se sources, did not vary significantly [38]. According to two previous studies, the color of breast meat was not significantly affected following supplementation with Se and vitamin E, either alone or in combination [17,39]. Finally, frozen storage already proved to be a factor affecting the color of breast fillets. In fact, similarly to the findings from this study, frozen breast meat tended to be darker, redder, and less yellow than the fresh counterpart after one [40] and two months of storage [54].

#### 3.1.3. WHC

The ability of meat to retain moisture, i.e., WHC, is a significant property controlled by meat industry and helpful to determine the yield and the overall quality during meat production and storage. In the present study, WHC was assessed in terms of drip loss and cooking loss in relation to the commercial size, the dietary treatment, and the storage (Table 2). Drip loss impairs the appearance of packaged meat, and, together with the cooking loss, they affect the juiciness and tenderness of the final cooked product. In the raw product, an increased drip loss may be related to the protein denaturation induced by a more or less intense post-mortem pH decline, leading not only to a decrease in the ability of proteins to bind water, but also an increase in permeability of cell membranes, resulting in an enhanced tissue exudation [55]. On the other hand, during cooking, the thermal processing induces further protein changes and water loss, leading inevitably to changes in sarcomere length and meat shrinkage [56].

Drip loss increased as the market age increased, although not significantly. In fact, light fillets showed lower loss than medium and heavy products (i.e., 2.76%, 2.88% and 2.82%, *p* > 0.05). By contrast, cooking loss slightly decreased, as breast muscles from light chickens experienced a water loss higher than meats from medium and heavy specimens (i.e., 23.45%, 23.41%, and 23.08%, *p* > 0.05). Light broilers were characterized by a lower pH of breast meat than medium and heavy breast muscles. Despite this, a slightly lower- and not higher-drip loss was found in light birds. This may be explained by the fact that the WHC may be more affected by muscle structural changes occurring during growth than the pH level, that not significantly varied with age according to our data [6,57]. Nevertheless, Berri and colleagues statistically demonstrated that the increase in cross sectional area of the muscle fiber observed during the growth of broiler, was not directly responsible for change in drip loss, that, conversely, appeared mostly correlated to the ultimate pH of meat [30]. However, a general tendency toward decreasing cooking losses with increasing pH of breast muscles was confirmed over the market age. Considering the diet, drip loss was significantly higher in standard fillets than the enriched counterpart (i.e., 3.44 and 2.77, *p* < 0.05). However, no significant differences were revealed between the two product categories with respect to the cooking loss. A flaxseed-based feeding is known to negatively affect the ability of meat to retain water as the increased susceptibility to lipid oxidation of muscle tissues results in the generation of free radicals which, in turn, cause protein degradation, and inevitably a deterioration of WHC, and drip loss of broiler meat [16,58]. However, the supplementation with antioxidants, such as Se and vitamin E, may considerably improve the oxidative status of meat and, consequently, delay the negative effects of lipid oxidation on meat technology [13,17,38]. This evidence could explain why in this study enriched meat showed a better WHC than the conventional counterpart. Frozen storage sensibly altered the WHC of breast fillets. In fact, both drip and cooking loss were significantly higher in frozen samples than fresh breast muscles (drip loss: 3.09% and 2.63%, *p* < 0.05; cooking loss: 23.94% and 22.68%, *p* < 0.05). The increase in drip loss and cooking loss observed in frozen/thawed breast meat was due to the tissue damage induced by the formation of ice crystals during the freezing process. In this respect, the alteration of cell structures because of protein denaturation may lead to a loss of ability to hold water [40,53,58,59]. According to the statistical model, the drip loss observed in breast meats was significantly affected by all the single variables investigated- but not their interaction-, whereas the cooking loss was significantly affected only by the freeze storage (Table 2).

With respect to the market age, findings from this study agreed with a previous work on commercial Italian broilers, which pointed out a non-specific trend of drip loss and a raising cooking loss in relation to the increasing commercial size [6]. However, in another trial conducted on breast meat from chickens with 35 and 42 days of life, the drip loss increased over time, whereas the cooking loss raised up to 38 days and then decreased by 42 days of life [34]. Considering the diet, several experiments already validated the positive influence of dietary antioxidants such as vitamin E and Se on the lipid stability—altered by the addition of FSM—and the WHC of poultry meat [13,38,60]. Nevertheless, the absence of any positive effect of vitamin E and/or Se on the WHC of breast meat was also observed [17]. In accordance with our results, the literature revealed that frozen storage consistently worsened the drip and cooking loss of breast meat, also when different times of storage were considered [40,54].

### 3.2. Sensorial Analysis

**Figure 2:**
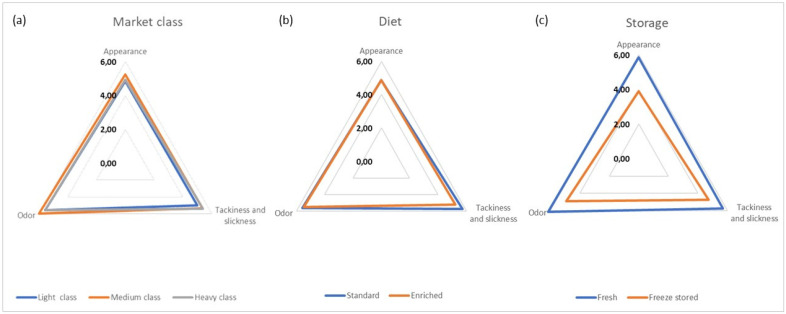
Sensory analysis of control and functional breast fillets from light, medium and heavy broilers at 24 h post-mortem and after freeze storage for 30 days (raw state). For every market class (**a**), data are reported in terms of mean ± standard deviation of *n* = 28 tastings of breast meat from broilers receiving standard (*n* = 14) and experimental (*n* = 14) feeding, both at 24 h post-mortem (*n* = 14) and following freeze storage (*n* = 14). For the dietary treatment (**b**), data are expressed as mean ± standard deviation of *n* = 42 tastings of meat from light (*n* = 14), medium (*n* = 14) and heavy (*n* = 14) broilers, both in fresh status (*n* = 21) and after freeze storage (*n* = 21). For fresh or freeze stored samples (**c**), data are expressed as mean ± standard deviation of *n* = 42 tastings of meat from light (*n* = 14), medium (*n* = 14) and heavy (*n* = 14) broilers receiving both standard (*n* = 21) and functional (*n* = 21) feed.

**Figure 3:**
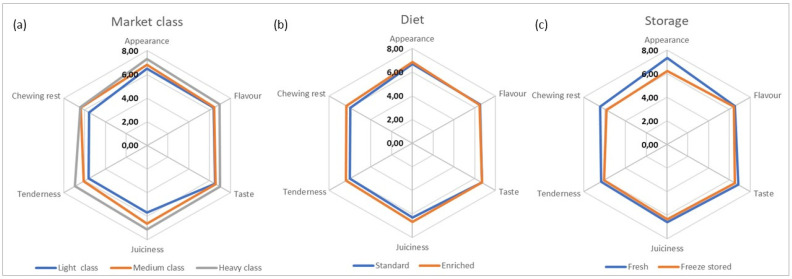
Sensory analysis of control and functional breast fillets from light, medium and heavy broilers at 24 h post-mortem and after freeze storage for 30 days (cooked state). For every market class (**a**), data are reported in terms of mean ± standard deviation of *n* = 28 tastings of breast meat from broilers receiving standard (*n* = 14) and experimental (*n* = 14) feeding, both at 24 h post-mortem (*n* = 14) and following freeze storage (*n* = 14). For the dietary treatment (**b**), data are expressed as mean ± standard deviation of *n* = 42 tastings of meat from light (*n* = 14), medium (*n* = 14) and heavy (*n* = 14) broilers, both in fresh status (*n* = 21) and after freeze storage (*n* = 21). For fresh or freeze stored samples (**c**), data are expressed as mean ± standard deviation of *n* = 42 tastings of meat from light (*n* = 14), medium (*n* = 14), and heavy (*n* = 14) broilers receiving both standard (*n* = 21) and functional (*n* = 21) feed.

The sensory traits of standard and enriched breast fillets from light, medium, and heavy chickens are shown in Figure 2 and Figure 3 and in Table S1.

For raw products, appearance, tackiness, and odor were not significantly different among products of broilers from different market classes (*p* > 0.05), as well as from birds fed enriched and conventional diet (*p* > 0.05). Overall, these attributes were evaluated between “neither pleasant/nor unpleasant” and “pleasant”, being around upper-middle scores (mean scores: 4.86–6.01). However, significant differences were observed in relation to the storage, as frozen-stored products were characterized by a lower sensory performance than fresh fillets (mean ratings: 3.89–4.91 vs. 5.71–6.14). In this respect, the storage was the fixed parameter that significantly impacted the sensory profile of breast fillets (*p* < 0.05) (Figure 2 and Table S1).

For cooked products, the appearance, juiciness, and chewing rest resulted significantly impacted by the market age, diet, and storage procedure (*p* < 0.05). The tenderness resulted significantly influenced by the market class (*p* < 0.05); while flavor and taste were not affected by any of the single variables investigated (*p* > 0.05). Interestingly, all the sensory notes tested in cooked products were significantly affected by the interaction between market class and diet (Table S1). With respect to the market class, flavor and taste notes slightly improved from “pleasant” to “very pleasant” going from light to heavy breasts (flavor: from 6.40 to 6.95 and taste: from 6.53 to 7.00, *p* > 0.05). Conversely, appearance, juiciness, tenderness, and chewing rest were between “slightly pleasant” and “pleasant” in light and medium products (mean scores: 5.49–6.83) and significantly (*p* < 0.05) improved in heavy products, which resulted between “pleasant” and “very pleasant” (mean scores: 6.43–7.30) (Figure 3, Table S1). In relation to the diet, the appearance (6.69 vs. 6.87, *p* > 0.05), flavor (6.52 vs. 6.46, *p* > 0.05), and taste (6.68 vs. 6.71, *p* > 0.05) were substantially similar in conventional and functional meats. Conversely, juiciness and chewing rest were more pleasant in functional than standard breasts (respectively, 6.33 and 6.44 vs. 5.80 and 5.81, *p* < 0.05), most likely due to the better WHC shown by functional fillets, as already described in paragraph 3.1.3. However, the tenderness degree was not significant different in standard and enriched products (5.88 vs.6.30, *p* > 0.05) (Figure 3, Table S1).

Based on the assumption that a level ≥ 5% of extruded FSM in the broiler diet could negatively affect the sensory traits of breast meat, including flavor and taste [36,61], the present study relied on the addition of 2.50–3.13% of extruded FSM to the diet just to avoid sensory alterations (i.e., rancid notes) in functional products. Additionally, the literature agreed to reveal non-significant modifications in the taste of cooked breasts due to different levels of Se and vitamin E supplemented in the diet of broilers [14]. However, contrasting conclusions were achieved for the other investigated notes. Indeed, previous studies highlighted no significant differences in the appearance, juiciness, and tenderness of cooked breasts from broilers fed FSM-supplemented diets [62,63]. On the other, the addition of different levels of Se and vitamin E resulted in higher hardness and chewiness of the breast samples [14]. Considering the storage effect, the overall appearance of products became less pleasant after freeze-storage and thawing (7.31 vs. 6.24, *p* < 0.05). Similarly, flavor (6.52 vs.6.45, *p* > 0.05) and taste (6.83 vs. 6.54, *p* > 0.05) were slightly worse in the stored products, while keeping pleasant to taste. Freeze-stored fillets showed also significantly lower performances for the appearance, the juiciness, and the chewing rest than the fresh counterpart (mean ratings: 7.31–6.44 vs. 5.81–6.03) (Figure 3, Table S1). This may be explained by an increased drip loss and cooking loss of frozen and thawed breast meat that, inevitably, was reflected in the sensory characteristics. Overall, our findings were consistent with the literature reporting a general deterioration of the sensory characteristics of broiler meat after freeze storage [11,64].

## 4. Conclusions

In the present study, the effect of market class, dietary enrichment with extruded FSM, Se, and vitamin E, and frozen storage, was evaluated on the technological and sensory profile of broiler breast meat with the help of a statistical multiple linear model.

Beside a normal color and pH characterizing every breast sample, functionalized muscles from heavy birds showed a better WHC than light fillets. However, frozen storage impared the technology of meat, as stored fillets were marked by consistent, but still normal, color variations, as they appeared darker, redder, and more yellow, as well as by a deteriorated WHC.

Concerning the sensory analysis, all the descriptors studied in cooked products were significantly affected by the mutual interaction between market class and diet. Additionally, in line with the results from technological analysis, notes such as juiciness and chewing rest improved considerably in enriched fillets from heavy broilers, being these descriptors strictly related to the WHC of meat. As expected, the frozen storage negatively altered the sensory properties of breast muscles, inducing a reduction of the scores of all investigated attributes.

Overall, findings from this study pointed out that fresh and functional breast fillets from heavy broilers had the best technological and sensorial traits, thus, confirming that both market class and diet are two relevant factors to consider for producing breast meat with higher technological and sensorial power.

## Figures and Tables

**Table 1:** Ingredients (%) and proximate composition (%) of control and functional diets. AME = apparent metabolizable energy. Retrieved from our previous work [7].

Ingredients		Starter	Grower 1	Grower 2	Finisher
		*Ingredients (%)*			
**Corn**	*Control*	45.70	48.53	55.27	56.88
	*Functional*	46.45	51.38	55.56	57.69
**Soybean meal**	*Control*	36.93	32.10	27.70	25.92
	*Functional*	36.40	32.22	27.73	25.65
**Wheat middlings**	*Control*	5.90	8.35	6.00	6.08
	*Functional*	4.00	4.00	4.00	4.00
**Sunflower meal**	*Control*	1.25	1.25	1.90	2.00
	*Functional*	1.00	1.00	1.00	1.00
**Oil, fat**	*Control*	4.20	5.15	5.70	5.95
	*Functional*	3.50	4.30	5.03	5.20
**Corn gluten meal**	*Control*	1.25	0.70	-	-
	*Functional*	1.32	0.55	-	-
**Extruded linseed meal**	*Control*	-	-	-	-
	*Functional*	2.50	2.50	3.13	3.13
**Vitamin E** **(100,000 UI/kg feed)**	*Control*	-	-	-	-
	*Functional*	0.22	0.23	0.23	0.23
**Selenium** **(2000 mg/kg feed)**	*Control*	-	-	-	-
	*Functional*	0.01	0.01	0.01	0.01
**Premix (Min. + Vit. + Enz. + Amin.)**	*Control*	4.77	3.92	3.43	3.17
	*Functional*	4.60	3.81	3.33	3.09
**Component**		*Proximate composition (%)*			
**Volume**	*Control*	100	100	100	100
	*Functional*	100	100	100	100
**AME (Kcal/kg)**	*Control*	2996	3094	3187	3214
	*Functional*	2991	3095	3184	3215
**Dry matter**	*Control*	89.34	89.20	89.00	89.00
	*Functional*	89.30	89.20	89.00	89.00
**Crude Protein**	*Control*	23.32	21.30	19.10	18.40
	*Functional*	23.30	21.30	19.20	18.40
**Crude Fiber**	*Control*	3.12	3.10	3.00	2.90
	*Functional*	3.20	3.10	2.90	2.90
**Ether extract**	*Control*	6.75	7.80	8.40	8.70
	*Functional*	6.90	7.70	8.70	8.90
**Ash**	*Control*	6.77	5.90	5.40	5.20
	*Functional*	6.80	6.10	5.50	5.30
**Lysine (digest)**	*Control*	1.28	1.15	1.02	0.96
	*Functional*	1.28	1.15	1.02	0.96
**Metionine+ Cysteine (digest)**	*Control*	0.95	0.87	0.80	0.75
	*Functional*	0.95	0.87	0.80	0.75
**Threonine (digest)**	*Control*	0.87	0.78	0.69	0.64
	*Functional*	0.87	0.78	0.69	0.64
**Calcium**	*Control*	1.00	0.90	0.80	0.70
	*Functional*	1.00	0.90	0.80	0.70
**Phosphorus**	*Control*	0.70	0.60	0.50	0.50
	*Functional*	0.70	0.60	0.50	0.50
Selenium (mg/Kg feed) *	*Control*	0.10	0.10	0.10	0.10
	*Functional*	0.40	0.40	0.40	0.40
**Vitamin E (mg/Kg feed)**	*Control*	84	70	70	70
	*Functional*	299	300	300	300

* Selenium: 2/3 sodium selenite and 1/3 organic selenium.

**Table 2:** Technological properties of control and functional breast fillets from light, medium, and heavy broilers at 24 h post-mortem and after freeze storage for 30 days. For every market class, data are reported in terms of mean ± standard deviation of *n* = 240 representative breasts from broilers receiving standard (*n* = 120) and experimental (*n* = 120) feeding, both at 24 h post-mortem (*n* = 120) and following freeze storage (*n* = 120). For the dietary treatment, data are expressed as mean ± standard deviation of *n* = 360 representative breast muscles from light (*n* = 120), medium (*n* = 120), and heavy (*n* = 120) broilers, both in fresh status (*n* = 180) and after freeze storage (*n* = 180). For fresh or freeze stored samples, data are expressed as mean ± standard deviation of *n* = 360 representative breasts from light (*n* = 120), medium (*n* = 120), and heavy (*n* = 120) broilers receiving both standard (*n* = 180) and functional (*n* = 180) feed.

Parameter		Market Class			Dietary Treatment		Storage		Source of Variation			
		Light Broiler	Medium Broiler	Heavy Broiler	Standard	Enriched	No Storage (Fresh Meat)	Frozen Storage (30d)	Market Class	Diet	Storage	Interaction
**pH**		5.81 ± 0.12	5.84 ± 0.05	5.92 ± 0.08	5.86 ± 0.11	5.85 ± 0.09	5.89 ± 0.10	5.82 ± 0.10	NS	NS	NS	NS
**Color**	L*	53.17 ± 1.38	54.28 ± 1.34	52.60 ± 1.04	53.47 ± 1.79	53.22 ± 1.04	54.13 ± 1.50	52.57 ± 1.59	NS	NS	NS	NS
	a*	2.47 ± 0.76 ^a^	2.55 ± 0.52 ^a^	2.97 ± 0.70 ^b^	2.67 ± 0.80	2.65 ± 0.59	2.04 ± 0.27 ^a^	3.28 ± 0.35 ^b^	*	NS	NS	* (M × D M × S D × S)
	b*	5.42 ± 0.75 ^a^	4.21 ± 0.44 ^b^	4.77 ± 0.28 ^c^	4.81 ± 0.77	4.79 ± 0.68	4.43 ± 0.49 ^a^	5.17 ± 0.73 ^b^	*	NS	*	* (M × D M × S D × S)
**WHC**	**Drip loss (%)**	2.76 ± 0.12	2.88 ± 0.19	2.82 ± 0.51	3.44 ± 0.33 ^a^	2.77 ± 0.31 ^b^	2.63 ± 0.21 ^a^	3.09 ± 0.25 ^b^	*	*	*	NS
	**Cooking loss (%)**	23.45 ± 0.89	23.41 ± 0.88	23.08 ± 0.76	23.28 ± 0.82	23.34 ± 0.89	22.68 ± 0.49 ^a^	23.94 ± 0.64 ^b^	NS	NS	*	NS

* Statistically significant (*p* < 0.05) and NS = non-significant by a multiple linear model. When significant relationships with fixed factor(s) and/or their interaction were found: different letters (^a,^ ^b,^ ^c^) in the same row indicate significantly different values among breast muscles from different market classes and/or diets (*p* < 0.05, by One-way ANOVA followed by post hoc Tukey’s HSD test or by *t*-test).

## Data Availability

Data is contained within the article or Supplementary Material.

## Supplementary Materials

The following supporting information can be downloaded at: https://www.mdpi.com/article/10.3390/foods11172567/s1. Table S1: Sensory analysis of control and functional breast fillets from light, medium, and heavy broilers at 24 h post-mortem and after freeze storage for 30 days (raw state). For every market class, data are reported in terms of mean ± standard deviation of *n* = 28 tastings of breast meat from broilers receiving standard (*n* = 14) and experimental (*n* = 14) feeding, both at 24 h post-mortem (*n* = 14) and following freeze storage (*n* = 14). For the dietary treatment, data are expressed as mean ± standard deviation of *n* = 42 tastings of meat from light (*n* = 14), medium (*n* = 14), and heavy (*n* = 14) broilers, both in fresh status (*n* = 21) and after freeze storage (*n* = 21). For fresh or freeze stored samples, data are expressed as mean ± standard deviation of *n* = 42 tastings of meat from light (*n* = 14), medium (*n* = 14), and heavy (*n* = 14) broilers receiving both standard (*n* = 21) and functional (*n* = 21) feed.
