# Supplementary material for: Effect of Dietary Enrichment with Flaxseed, Vitamin E and Selenium, and of Market Class on the Broiler Breast Meat—Part 2: Technological and Sensorial Traits
Source: Foods. 2022 Aug 25;11(17):2567. doi: 10.3390/foods11172567 (PMC9455164; doi:10.3390/foods11172567)
Supplement: Supplementary file 1 [file foods-11-02567-s001.zip › foods-1876989-supplementary.pdf]

## Supplementary Material

**Table S1.** Sensory analysis of control and functional breast fillets from light, medium and heavy broilers at 24 h post-mortem and after freeze storage for 30 days (raw state). For every market class, data are reported in terms of mean ± standard deviation of *n*=28 tastings of breast meat from broilers receiving standard (*n*=14) and experimental (*n*=14) feeding, both at 24h post-mortem (*n*=14) and following freeze storage (*n*=14). For the dietary treatment, data are expressed as mean ± standard deviation of *n*=42 tastings of meat from light (*n*=14), medium (*n*=14) and heavy (*n*=14) broilers, both in fresh status (*n*=21) and after freeze storage (*n*=21). For fresh or freeze stored samples, data are expressed as mean ± standard deviation of *n*=42 tastings of meat from light (*n*=14), medium (*n*=14) and heavy (*n*=14) broilers receiving both standard (*n*=21) and functional (*n*=21) feed.

| Attribute | Market Class | | | Dietary Treatment | | Storage | | Source of Variation | | | |
|---|---|---|---|---|---|---|---|---|---|---|---|
| | Light Broiler | Medium Broiler | Heavy Broiler | Standard | Enriched | No Storage (Fresh Meat) | Freeze Storage (30d) | Market Class | Diet | Storage | Interaction |
| *Raw products* | | | | | | | | | | | |
| **Appearance** | 4.83±1.10 | 5.26±0.62 | 4.93±0.92 | 4.87±1.01 | 4.86±1.03 | 5.84±0.28$^{a}$ | 3.89±0.17$^{b}$ | NS | NS | * | NS |
| **Tackiness/ slickness** | 4.99±0.44 | 5.36±0.59 | 5.41±0.53 | 5.73±0.60 | 5.22±0.55 | 5.71±0.37$^{a}$ | 4.74±0.21$^{b}$ | NS | NS | * | NS |
| **Odour** | 5.57±0.85 | 6.01±0.25 | 5.58±0.54 | 5.59±0.74 | 5.46±0.63 | 6.14±0.32$^{a}$ | 4.91±0.20$^{b}$ | NS | NS | * | * (M×D) |
| *Cooked products* | | | | | | | | | | | |
| **Appearance** | 6.49±0.94$^{a}$ | 6.83±0.45$^{a}$ | 7.30±0.23$^{b}$ | 6.69±0.67 | 6.87±0.64 | 7.31±0.16$^{a}$ | 6.24±0.53$^{b}$ | * | * | * | * (M×D) |
| **Flavour** | 6.40±0.23$^{a}$ | 6.42±0.19 | 6.95±0.44 | 6.52±0.22 | 6.46±0.22 | 6.52±0.17 | 6.45±0.26 | NS | NS | NS | * (M×D) |
| **Taste** | 6.53±0.19 | 6.56±0.20 | 7.00±0.47 | 6.68±0.32 | 6.71±0.39 | 6.83±0.36 | 6.54±0.19 | NS | NS | NS | * (M×D) |
| **Juiciness** | 5.68±0.25$^{a}$ | 6.30±0.61$^{a}$ | 7.14±0.31$^{b}$ | 6.15±0.68 | 6.59±0.71 | 6.71±0.30$^{a}$ | 6.03±0.44$^{b}$ | * | * | * | NS |
| **Tenderness** | 5.49±0.36$^{a}$ | 5.85±0.43$^{b}$ | 6.93±0.46$^{c}$ | 5.88±0.72 | 6.30±0.71 | 6.33±0.78 | 5.80±0.57 | * | NS | NS | * (M×D) |
| **Chewing rest** | 5.57±0.69$^{a}$ | 6.35±0.53$^{b}$ | 6.43±0.23$^{b,c}$ | 5.96±0.58$^{a}$ | 6.32±0.69$^{b}$ | 6.44±0.38$^{a}$ | 5.81±0.73$^{b}$ | * | * | * | * (M×D) |

*Statistically significant ($p<0.05$) and NS=non-significant by a multiple linear model. When significant relationships with fixed factor(s) and/or their interaction were found: different letters (a,b,c) in the same row indicate significantly different values among breast muscles from different market classes and/or diets ($p < 0.05$, by One-way ANOVA followed by post hoc Tukey's HSD test or by t-test).
